# FAMILY GUIDANCE WEAKENS AN EFFECT OF OLD PEOPLE’S EDUCATIONAL ATTAINMENT ON EHEALTH LITERACY BY INTERNET EXPERIENCE

**DOI:** 10.1093/geroni/igad104.3063

**Published:** 2023-12-21

**Authors:** Jingxuan Wu, Huamao Peng, Zhiyu Fan, Heng Zhao

**Affiliations:** Beijing Normal University, Beijing, Beijing, China (People’s Republic); Beijing Normal University, Beijing, Beijing, China (People’s Republic); Beijing Normal University, Beijing, Beijing, China (People’s Republic); Beijing Normal University, Beijing, Beijing, China (People’s Republic)

## Abstract

**Background:**

In the emerging era of digitalization, eHealth literacy is of great importance in older adults’ health and social adaptation. Objective: Explore the effect of educational attainment on eHealth literacy among older adults and examine the mediating role of Internet experience and the moderating role of guidance on the Internet from family members to reveal the underlying mechanism. Method: Four hundred and ninety-one older adults (aged 59–76 years old, Mage = 65.69±4.41) completed the eHealth Literacy Scale (eHEALS) and Internet Experience among Older Adults Scale (IEOAS) and reported their educational attainment and the frequency of family members guiding them on how to use the Internet.

**Results:**

eHealth literacy of older adults with lower educational attainment did not reach an intermediate level (M = 2.46±1.17). It was significantly lower than that of older adults with higher educational attainment whose scores were beyond the intermediate level (M = 3.43±0.90). Internet experience mediated the effect of older adults’ educational attainment on eHealth literacy. This mediation model was moderated by family members’ guidance on the Internet.

**Discussion:**

our results have implications for understanding how educational attainment reinforced the inequality in eHealth literacy among older adults and how to address this gap. Specifically, If older adults with lower educational attainment are given guidance on Internet use by their family members frequently, they will no longer be at a disadvantage in accumulating Internet experience and further cultivate eHealth literacy to search for, distinguish, and take advantage of health information on the Internet well.

